# Multi-modal Malignancies in Cowden Syndrome: Diagnostic Challenges in a Suspected Case From a Low-Resource Setting

**DOI:** 10.7759/cureus.91827

**Published:** 2025-09-08

**Authors:** Jawayria Sajid, Rohma Qureshi, Hamza Ahmad, Asad ur Rehman Qureshi, Ahsan Shafiq

**Affiliations:** 1 Medical Oncology, Shalamar Medical and Dental College, Lahore, PAK; 2 General Medicine, Shalamar Institute of Health Sciences, Lahore, PAK; 3 General Surgery, Shalamar Institute of Health Sciences, Lahore, PAK; 4 Otolaryngology - Head and Neck Surgery, Combined Military Hospital, Okara, PAK; 5 Surgical Oncology, Shaukat Khanum Memorial Cancer Hospital and Research Centre, Lahore, PAK; 6 Surgical Oncology, Shalamar Medical and Dental College, Lahore, PAK

**Keywords:** breast cancer, cowden syndrome, hereditary cancer syndromes, low-middle income country, low-resource setting, multiple primary tumors, papillary thyroid carcinoma, pten mutation, renal cell carcinoma

## Abstract

Cowden syndrome (CS), a rare autosomal dominant disorder caused by mutations in the PTEN tumor suppressor gene, predisposes individuals to a wide range of malignancies, including breast, thyroid, endometrial, and renal cancers. This report presents a case of a 69-year-old woman with a history of papillary thyroid carcinoma, recently diagnosed invasive ductal carcinoma of the breast, and incidental clear cell renal cell carcinoma (RCC) - clinically pointing toward the diagnosis of CS. Genetic testing and endoscopic evaluations were not possible, as the case occurred in the setting of a developing country, with limited resources and financial constraints.

This case underscores the importance of early recognition of hereditary cancer syndromes in patients with multiple malignancies, as well as the need for comprehensive genetic counseling, surveillance, and tailored treatment strategies. A multidisciplinary approach involving oncology, surgery, radiology, and genetics is crucial in managing the complex clinical presentation of patients with CS. The case also highlights the challenges faced when establishing a formal diagnosis in resource-constrained settings. These challenges are related not only to limited resources, but also to patient compliance, health literacy, and access to healthcare services.

## Introduction

Cowden syndrome (CS) is a hallmark disorder of the PTEN hamartoma tumor syndrome (PHTS) spectrum, caused by germline pathogenic variants in the PTEN gene. PHTS includes CS, Bannayan-Riley-Ruvalcaba syndrome (BRRS), and Proteus-like syndrome, with affected individuals at elevated risk for various malignancies, particularly breast, thyroid, and renal cancers, as well as endometrial and colorectal cancers, and melanoma [1,2]. Lifetime cancer risks in PHTS are high, ranging from 85% to 89% for any cancer, with up to 85% for breast cancer in women [1,2]. The PTEN gene, located on chromosome 10q23, regulates tumor suppression by negatively controlling the PI3K/AKT pathway [3,4]. Mutations or loss of function in PTEN are linked to several cancers, including glioblastoma, lung, breast, and prostate cancers. More than 3,000 cases of CS have been described, though underdiagnosis is common due to phenotypic variability [2,5]. In resource-limited settings, comprehensive germline testing is often impractical. Thus, an operational diagnosis of CS was adopted based on clinical reasoning, the pattern of malignancies, and the need to guide surveillance and familial counseling. This case underscores the diagnostic challenges in atypical presentations of cancer predisposition syndromes and highlights the need for pragmatic decision-making in low-resource environments. Clinicians and radiologists should remain alert when encountering multiple malignancies in a single patient [6].

## Case presentation

A 69-year-old woman presented with a one-year history of nipple retraction, intermittent abdominal pain, and anorexia. She had a past history of ischemic heart disease (well controlled) and a total thyroidectomy in 2013 for multinodular goiter, which was later confirmed to be papillary thyroid carcinoma (treated). She received radioactive iodine ablation and has remained on thyroxine, with no recurrence. Family history includes unspecified gastrointestinal (GI) malignancy in her mother.

**Figure 1:**
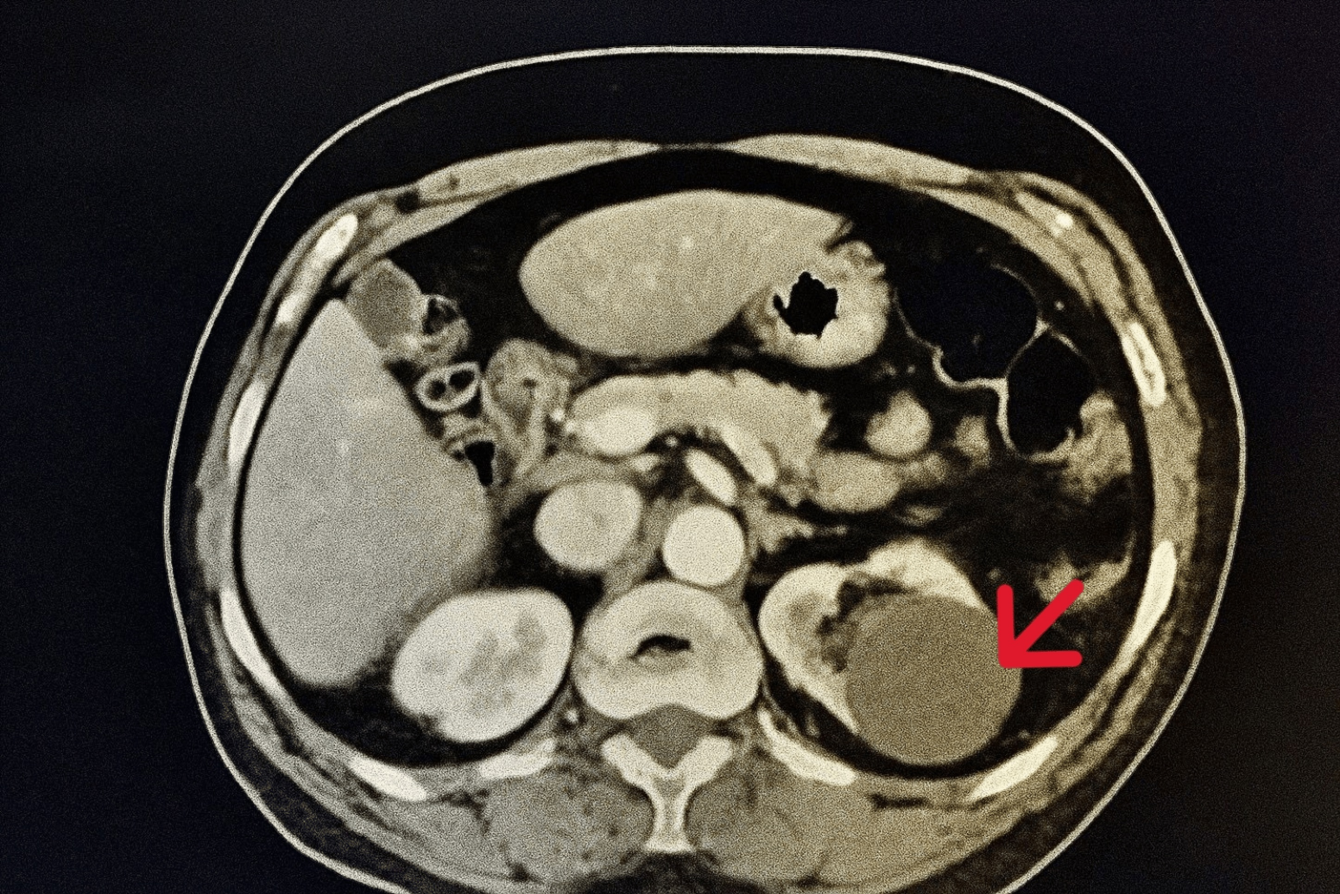
CT-Scan Abdomen Showing Left Renal Mass (RCC) The red arrow indicates a renal mass suggestive of renal cell carcinoma (RCC).

Systemic imaging with CT of the chest, abdomen, and pelvis (CT CAP) incidentally identified a solid-cystic lesion in the left kidney, which was subsequently biopsied and confirmed as clear cell renal cell carcinoma (RCC), Grade I. A bone scan showed a sclerotic lesion at T7, which was determined to be non-metastatic. A CT image showing the renal mass is shown in Figure 1.

**Figure 2:**
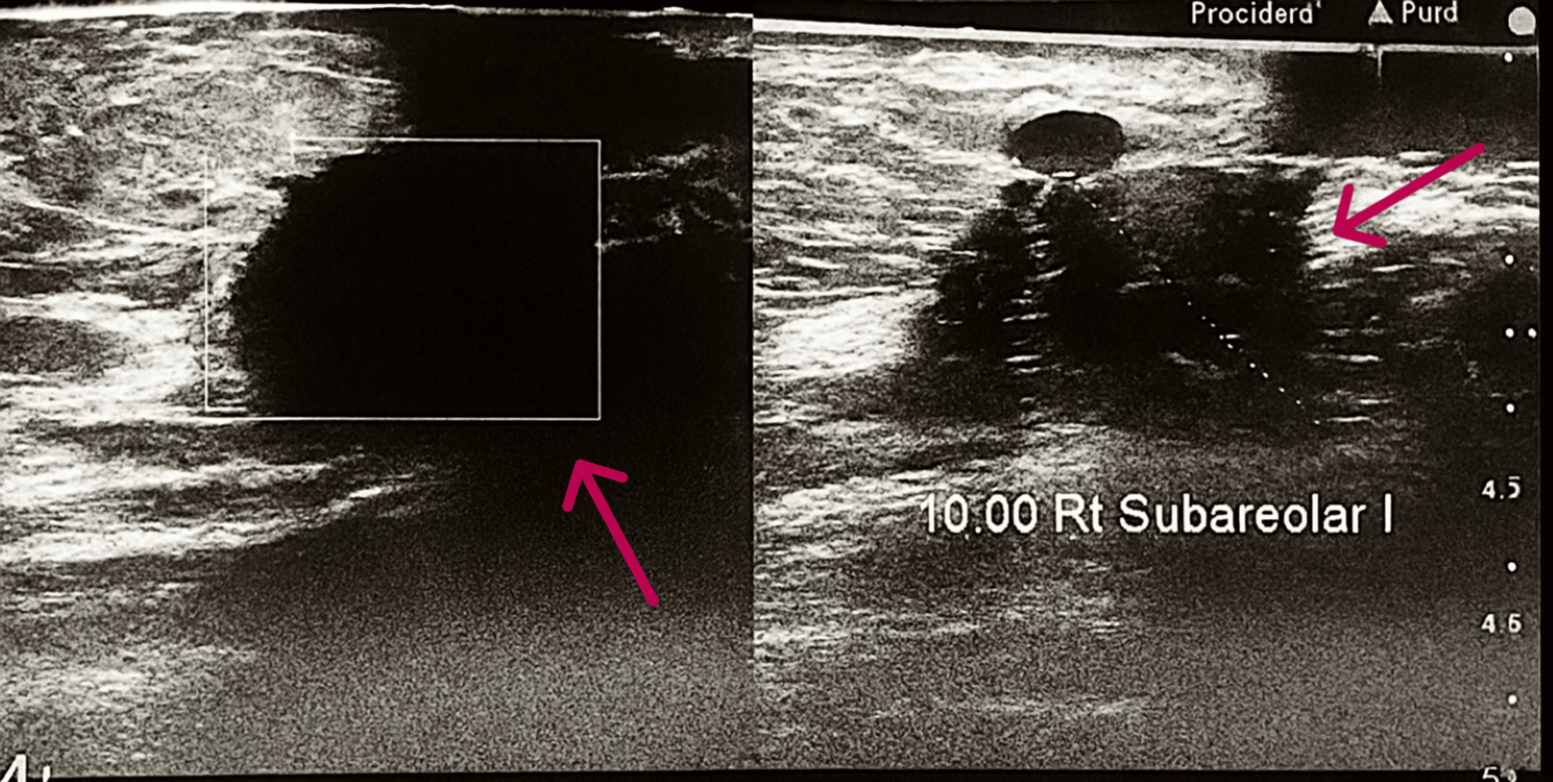
Right Breast Mammography Showing Spicualted Opacity A spiculated opacity is noted in the right outer quadrant of the breast, causing local architectural distortion. No suspicious microcalcifications are seen. Mild skin thickening and nipple retraction are noted. Correlative breast ultrasound shows an irregular hypoechoic lesion with infiltrative margins at the 10 o’clock peri-areolar position, measuring 23 × 23 mm. Significant vascularity on color Doppler and intense posterior shadowing look suspicious.

**Figure 3:**
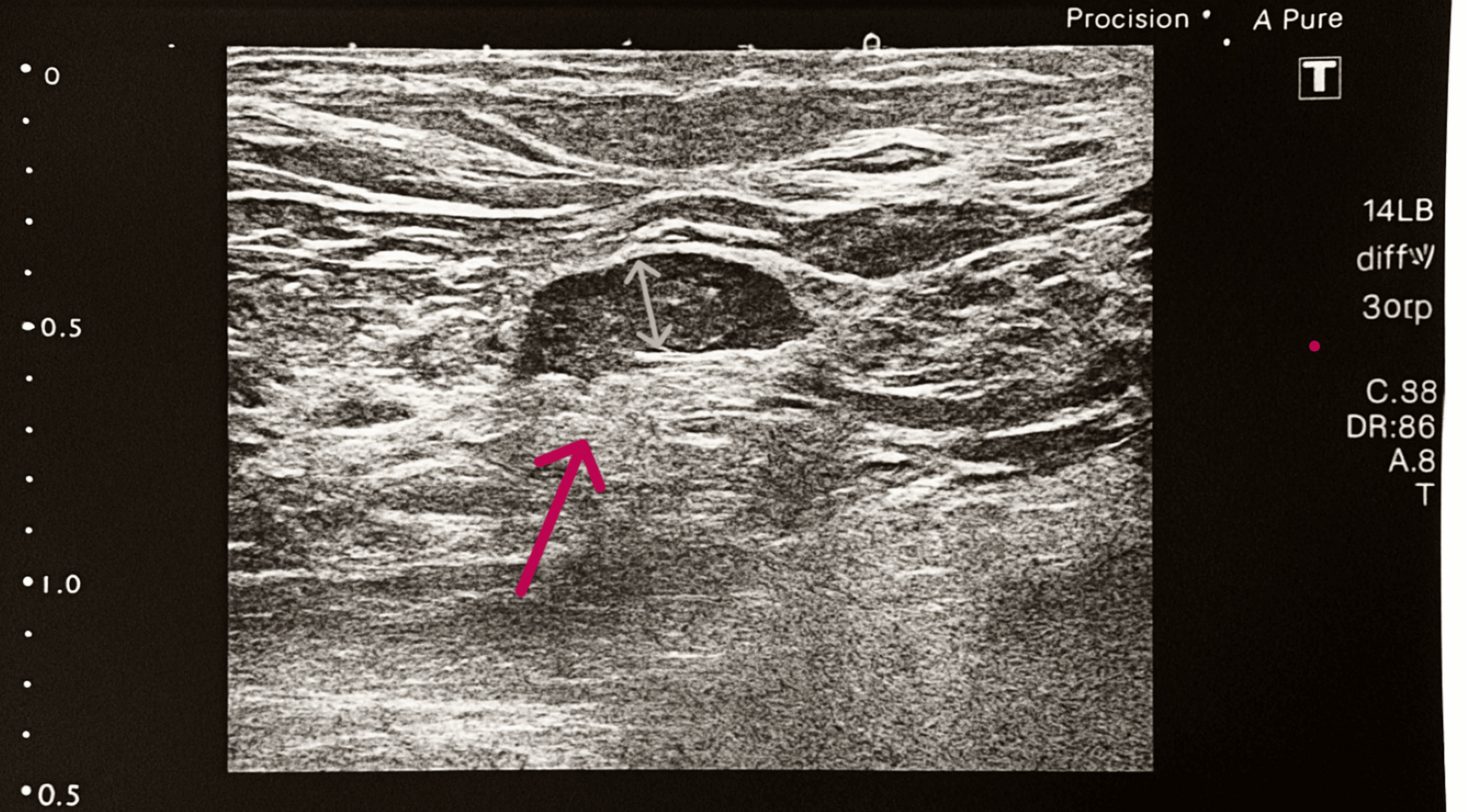
Right Breast Mammography Showing Lymphadenopathy A few suspicious, rounded-looking axillary lymph nodes are seen, with lost fatty hila; the largest measures 17.5 × 8.5 mm (red arrow).

The patient presented with a spiculated opacity in the right upper outer quadrant on mammogram, categorized as BIRADS (Breast Imaging Reporting and Data System) V. Ultrasound revealed a hypoechoic lesion with suspicious axillary lymph nodes. A core biopsy from Shaukat Khanum Memorial Cancer Hospital Laboratory confirmed invasive ductal carcinoma, Grade III, that was estrogen receptor (ER)-positive, progesterone receptor (PR)-positive, human epidermal growth factor receptor 2 (HER2)-negative, with a Ki-67 index of 20%. A mammographic image of the breast mass and associated lymphadenopathy is shown in Figures 2-3.

**Figure 4:**
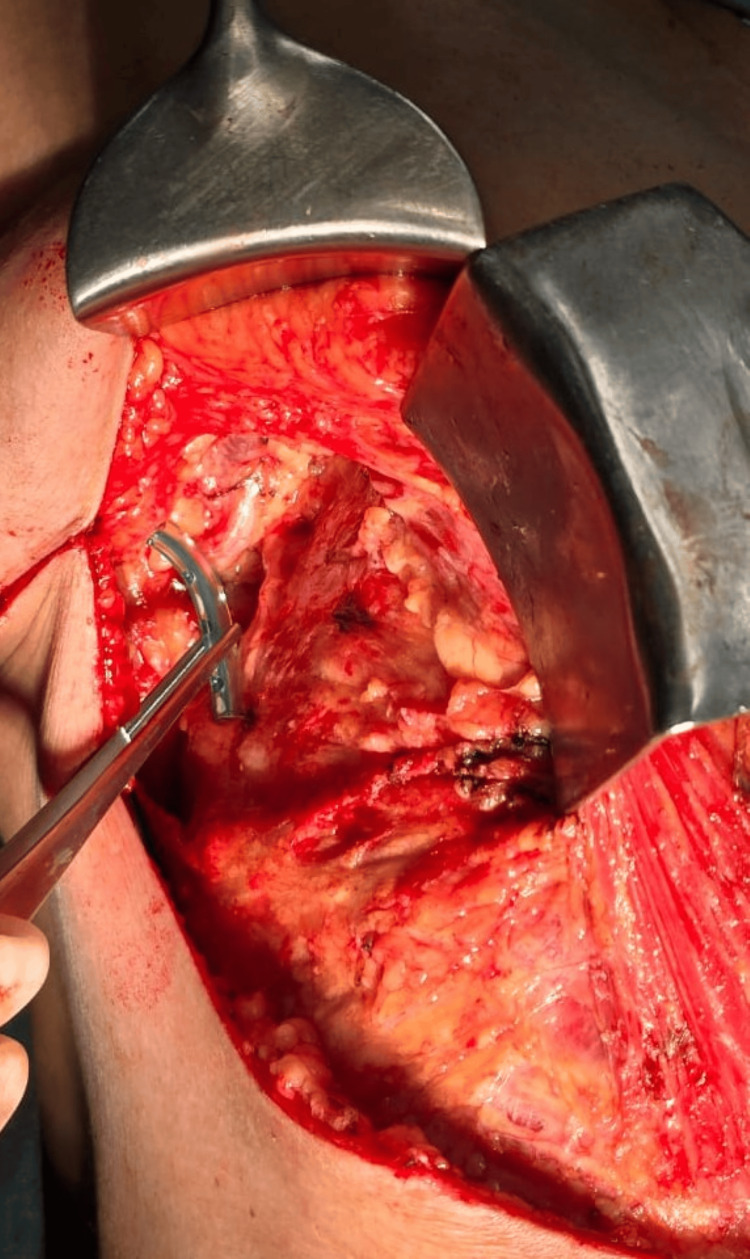
Intraoperative Image of a Modified Radical Mastectomy

**Figure 5:**
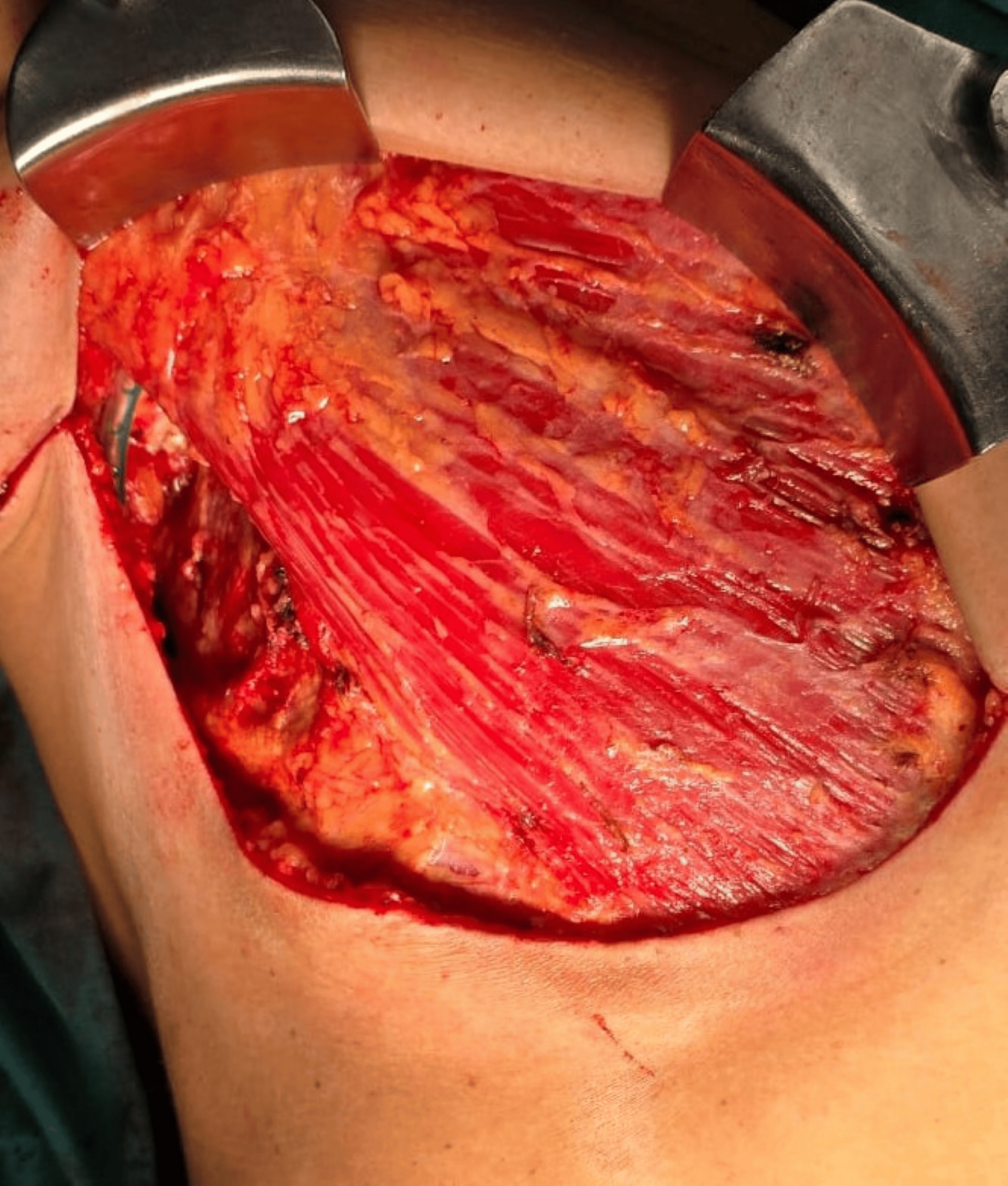
Intraoperative Image of a Modified Radical Mastectomy

**Figure 6:**
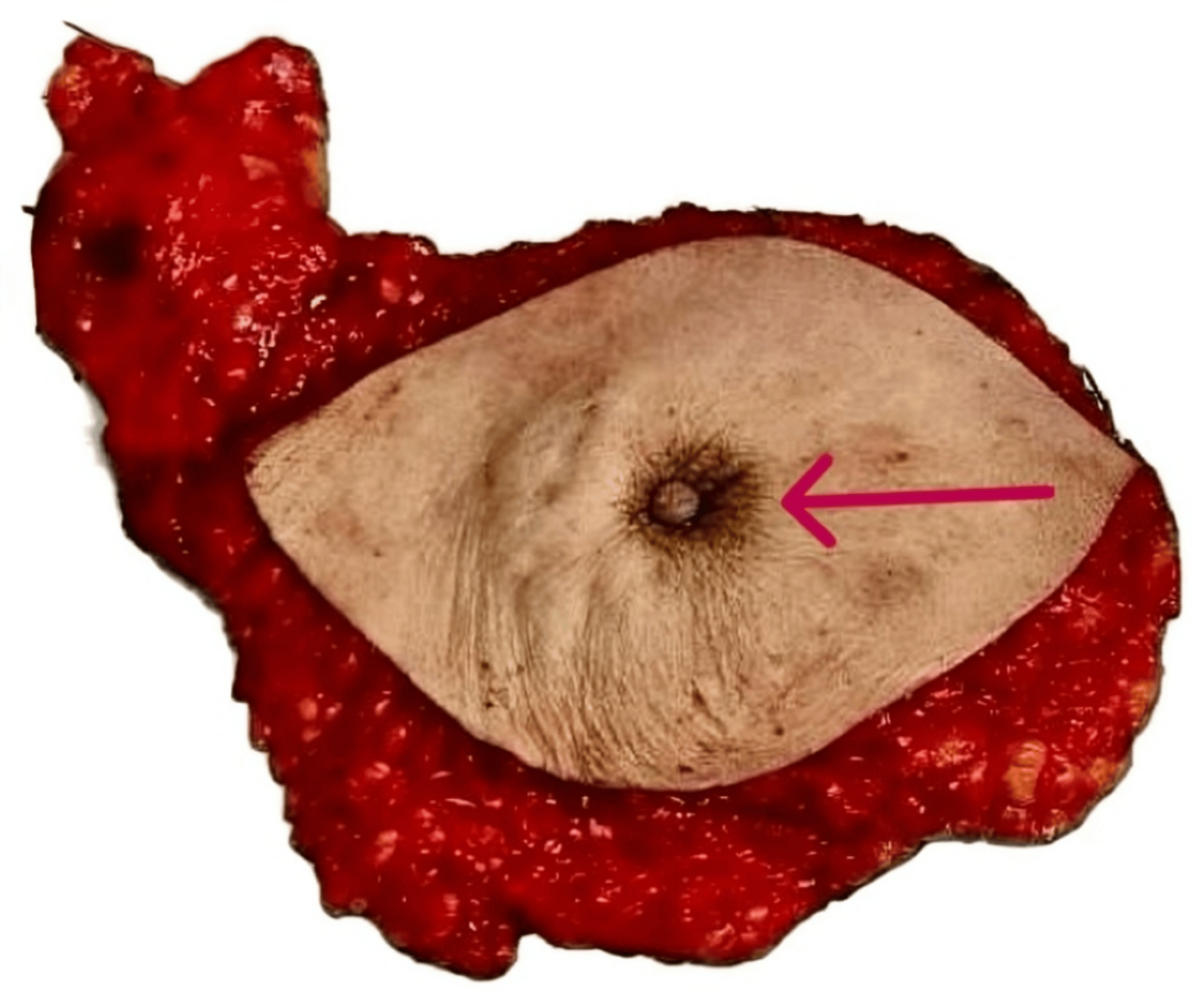
MRM Specimen Showing Nipple Retraction The red arrow indicates the point of interest. MRM, Modified radical mastectomy

**Figure 7:**
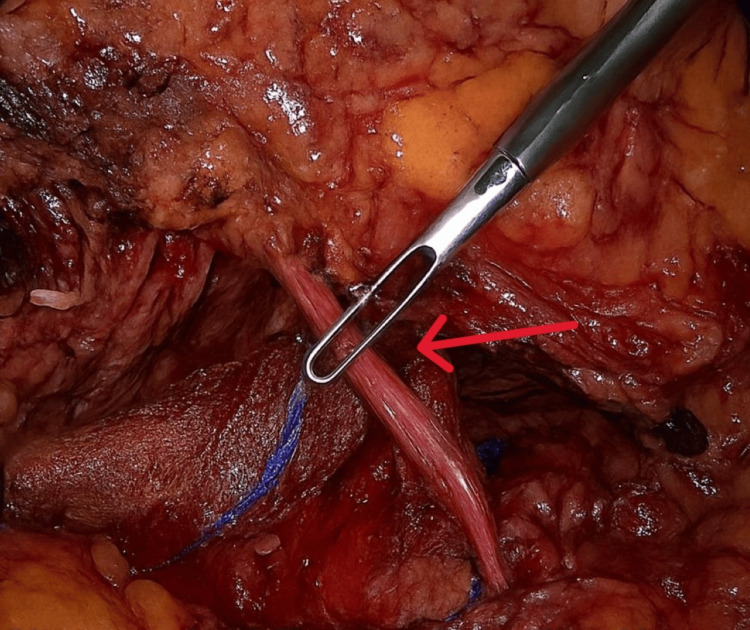
Laparoscopic Left Radical Nephrectomy Showing Clipping of the Ureter The red arrow indicates the point of interest.

**Figure 8:**
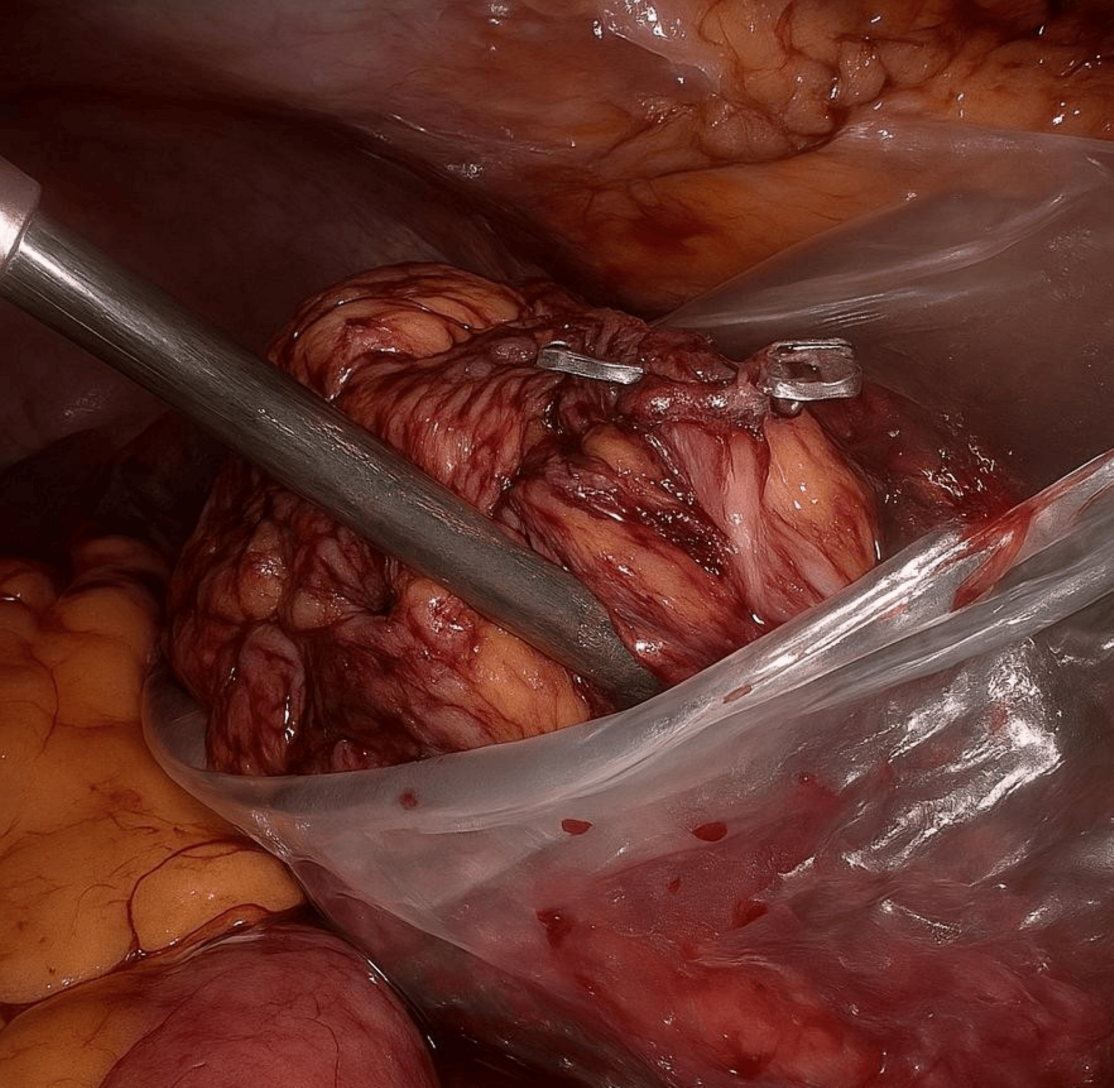
Laparoscopic Left Radical Nephrectomy Showing Removal of Specimen via Endo Bag

Given the presence of multiple primary malignancies, along with a history of thyroid cancer, CS was suspected. Letrozole, which had been initiated at the first clinical encounter, was discontinued. Following a multidisciplinary team (MDT) discussion, the patient underwent a right modified radical mastectomy and a laparoscopic left radical nephrectomy. Images from the modified radical mastectomy and left radical nephrectomy procedures for this patient are shown in Figures 4-8.

The patient’s treatment and surveillance plan closely adheres to the National Comprehensive Cancer Network (NCCN) 2025 guidelines. Following surgical management of breast cancer, she has completed eight cycles of the CMF (cyclophosphamide, methotrexate, 5-fluorouracil) chemotherapy regimen. She is scheduled to undergo chest wall and regional nodal radiation therapy. Upon completion of adjuvant chemotherapy, she will be initiated on aromatase inhibitors, appropriate for her postmenopausal status, for a duration of five years due to her ER+, PR+ breast cancer. Hormonal therapy can be extended further if well- tolerated. During this period, routine follow-up will include regular history and physical examinations and annual mammography. 

RCC was Stage I; thus, following her radical nephrectomy, she will be scheduled for regular surveillance. CT scans of the chest and abdomen will be performed to monitor for RCC. The first follow-up CT will be done six months post-surgery, with subsequent imaging conducted annually in alignment with NCCN 2025 recommendations. 

## Discussion

The patient presented with a rare triad of malignancies: papillary thyroid carcinoma, invasive ductal carcinoma of the breast, and clear cell RCC. While CS - part of the PTEN hamartoma tumor spectrum - is the most recognized cancer predisposition syndrome linking thyroid and breast malignancies, this patient’s clinical picture deviates from the classic phenotype.

Notably, she lacks many of the hallmark mucocutaneous lesions and macrocephaly typically seen in CS. Similarly, her renal tumor histology (clear cell RCC) is less commonly associated with the syndrome. However, alternative hereditary cancer syndromes (e.g., Li-Fraumeni syndrome, hereditary leiomyomatosis and renal cell cancer (HLRCC), Birt-Hogg-Dubé syndrome (BHDS), and von Hippel-Lindau disease (VHL)) fail to account for this specific constellation of neoplasms or are unlikely given the clinical and family history.

CS is clinically heterogeneous, but combinations of breast, thyroid, and renal cancers - as in this case - are strongly suggestive of the diagnosis. According to the International Cowden Consortium criteria and NCCN guidelines, this patient fulfills one major criterion (breast cancer) and three minor criteria (RCC, papillary thyroid carcinoma, and nodular goiter) [1,3].

Breast cancer is the most frequent malignancy in CS, typically ER+/PR+ and HER2-, as in our patient [5,7]. Papillary thyroid carcinoma is the most common thyroid subtype in CS, while renal cancer - particularly clear cell RCC - is increasingly recognized as part of the syndrome's phenotypic spectrum [8,9].

Recent radiological studies emphasize the importance of recognizing early imaging signs, like architectural distortion in the breast or mixed cystic-solid renal lesions, in CS [6]. The patient’s imaging findings align well with reported patterns.

Genetic testing for PTEN mutations is the gold standard for diagnosis, aiding both in confirmation and in guiding screening for family members [2,3,10]. Bibliometric studies also show a rise in CS-related publications, particularly in understanding PTEN's molecular and therapeutic implications [11].

This case reinforces the importance of multidisciplinary care - involving radiology, oncology, surgery, and genetics - in the diagnosis and management of hereditary cancer syndromes like CS, while paying close attention to the diagnostic and surveillance challenges faced in resource-constrained settings. Tables 1-2 show guidelines adapted from the official NCCN Version 2.2025 for screening and surveillance in these cases [12].

**Table 1:** NCCN-Adapted Guidelines Version 2.2025: Cowden Syndrome (CS)/PTEN Hamartoma Tumor Syndrome (PHTS) Management Guidelines and table adapted from NCCN [12]. This table has been independently adapted by the authors, based on clinical concepts from available NCCN guidelines. All content has been reworded and reformatted for original presentation; no copyrighted material has been directly reproduced. NCCN, Nation Comprehensive Cancer Network

System/Organ	Suggested Monitoring & Timing
Endometrium	Begin evaluations for uterine lining cancer at age 35. Educate patients to report unusual menstrual or postmenopausal bleeding, and encourage tracking cycles. Symptoms should prompt investigation with an endometrial biopsy, not just imaging. Routine biopsies may be repeated every 1-2 years if deemed appropriate. Ultrasound is not a reliable tool for premenopausal patients due to normal variations in endometrial thickness but may have limited use postmenopause at the provider’s discretion. Prophylactic hysterectomy can be discussed after childbearing is complete; ovaries do not need to be removed unless another indication exists. Provide support around the psychological and lifestyle effects of surgical decisions.
Kidneys	Begin ultrasound screening for kidney tumors at age 40 and repeat every 1-2 years.
Neurological	In children, assess for developmental or motor delays at diagnosis. Brain MRI should be performed if neurological concerns are present.
Skin	Regular dermatology checks are advised, ideally once a year, due to a higher risk of melanocytic and other skin lesions.
Thyroid	Begin neck ultrasounds by age 7. In children with a 50% chance of inheriting a known gene mutation, screening can start even if genetic testing is postponed until adulthood.
Reproductive Planning	For personalized guidance about fertility, cancer risk, and family planning, consult genetic risk assessment resources.
Family Risk Assessment	Relatives should undergo risk assessment using established genetic counseling frameworks.

**Table 2:** NCCN Guidelines, Version 2.2025: Cowden Syndrome (CS)/PTEN Hamartoma Tumor Syndrome (PHTS) Management Guidelines obtained from the official website of NCCN [12]. This table has been independently adapted by the authors, based on clinical concepts from publicly available NCCN guidelines. All content has been reworded and reformatted for original presentation; no copyrighted material has been directly reproduced. NCCN, National Comprehensive Cancer Network

Focus Area	Recommended Approach
General Health	Due to the condition’s complexity, referral to clinicians familiar with CS/PHTS is strongly advised. Comprehensive physical exams should begin at age 18 or 5 years before the earliest known cancer in the family - whichever is sooner. Emphasize patient education about early warning signs of cancer.
Female Breast	Encourage awareness of breast changes starting at 18. Begin clinical exams every 6-12 months from age 25 or earlier if there's a family history. Annual mammograms and MRIs (with and without contrast) should begin at age 30 or 10 years before the youngest breast cancer case in the family. Screening may be adapted for women over 75 depending on individual health. Continue imaging even after breast cancer treatment if both breasts haven’t been removed. Offer risk-reducing surgery (RRM) to those with pathogenic variants - decisions should reflect family history and personal risk. Pre-surgical counseling must cover expected benefits, reconstructive choices, and future risk. Emotional support and quality-of-life factors should always be addressed.
Colon/Rectum	Begin colonoscopy by age 35, or earlier if there is a strong family history (e.g., diagnosis under 40 in a first-degree relative). Rescreen every 5 years, or more frequently if symptoms develop or polyps are found.

The revised clinical criteria for PHTS include major and minor features. Major criteria include breast, endometrial, and follicular thyroid cancers; GI hamartomas (excluding hyperplastic polyps); Lhermitte-Duclos disease; macrocephaly (≥58 cm in females and ≥60 cm in males); macular pigmentation of the glans penis; and multiple mucocutaneous lesions (e.g., trichilemmomas, acral keratoses, neuromas, or oral papillomas). Minor criteria include autism, colon cancer, esophageal glycogenic acanthosis, lipomas (≥2), intellectual disability (IQ ≤75), RCC, testicular lipomatosis, papillary thyroid cancer or variants, thyroid structural lesions, and vascular anomalies [13].

A diagnosis is made in individuals with three or more major criteria (one must be macrocephaly, Lhermitte-Duclos disease, or GI hamartomas), or two major plus three minor criteria. In families with a known PTEN mutation or confirmed case, diagnosis requires either two major, one major plus two minor, or three minor criteria [13,14].

A study on PTEN-wildtype CS, CS-like syndromes, and BRRS examined genetic causes of “missing heritability” in patients without PTEN mutations. Only 8% had pathogenic variants in high-penetrance cancer genes; many had variants of uncertain significance (VUS), particularly in MET. Some mutations involved non-cancer-related genes, suggesting alternative genetic or epigenetic mechanisms. Patients with germline variants more often had multiple primary tumors [13,14].

While expanding gene panels showed limited diagnostic benefit, clinical features aligned with gene-specific cancer risks. The study emphasizes gene-based management for mutation-positive individuals and CS/BRRS guidelines for PTEN-wildtype patients with strong phenotypes. Further research is needed into broader genomic and epigenetic mechanisms [15]. Genetic counseling and testing, especially for those with a family history, remain vital for risk assessment and personalized care in CS [16,17].

PTEN loss in clear cell renal carcinoma suggests possible loss of heterozygosity, while its retention in papillary renal carcinoma indicates a likely sporadic origin. CS, a subset of PHTS, is associated with increased risk of renal, breast, and thyroid cancers, with most related tumors showing PTEN loss [18]. This activates the PI3K/AKT/mTOR pathway, offering a rationale for targeted therapies. In PTEN-deficient breast cancers, targeted agents such as AKT inhibitors and mTOR inhibitors may be beneficial, particularly when standard treatments fail [19]. Renal cancers in CS may also benefit from mTOR inhibitors, though outcomes vary [20]. Surgical treatment follows standard oncologic protocols - mastectomy for breast cancer, thyroidectomy for thyroid malignancies, and standard nephrectomy for renal tumors - with targeted therapies enhancing individualized care [20]. Vigilant surveillance remains essential for early detection. CS can affect multiple organ systems, including the breast, thyroid, kidneys, GI tract, and skin, with variable risk for malignancy [12].

This case exemplifies the diagnostic and systemic challenges faced in low- and middle-income countries (LMICs). While the clinical presentation is strongly suggestive of CS, confirming the diagnosis through genetic testing is often unattainable due to financial constraints. Testing for PTEN mutations, typically included in multi-gene panels, is prohibitively expensive for patients with average income, and public hospitals often lack the funding to subsidize such tests [13,14]. In a study related to hereditary breast cancers in Pakistan, the authors discuss strategies implemented to overcome these barriers, including provider education and the integration of genetic services into existing clinical workflows [20]. Another report also underscores the challenges in diagnosing CS due to its variable presentation, and emphasizes the need for awareness and access to genetic services for appropriate management [19].

In our case, GI malignancies and hamartomatous lesions were considered as part of the differential, given the known spectrum of CS. Systemic imaging (CT chest, abdomen, and pelvis) did not reveal any suspicious GI lesions, and while upper GI endoscopy or colonoscopy would have been ideal for comprehensive assessment, these options were discussed with the patient and her family, but ultimately not pursued due to financial and logistic barriers. Importantly, the decision was not a result of insufficient counseling or patient reluctance; rather, priority was given to the management of the two confirmed synchronous malignancies and the patient’s existing comorbidities. Family counseling was undertaken, and first-degree relatives were informed of the potential hereditary nature of CS. In the absence of genetic confirmation, surveillance based on the NCCN major and minor clinical criteria was recommended, particularly focusing on breast, thyroid, and renal screening. Efforts are ongoing within our institution to link high-risk families with subsidized or charitable resources for genetic testing and surveillance, which may provide more equitable access in the future [12,15].

Moreover, recommending additional evaluations - such as upper GI endoscopy or colonoscopy to assess for hamartomas or GI malignancies - poses both logistical and psychological hurdles. Limited health literacy, restricted access to specialized care, and financial insecurity make it difficult for patients to pursue these investigations [15,19]. In resource-limited settings, confirming complex hereditary cancer syndromes like CS becomes a significant burden not only for patients but also for the healthcare system, often leading to delays in diagnosis and suboptimal surveillance for associated malignancies [17,18].

## Conclusions

This case illustrates the complex diagnostic and management challenges posed by CS, particularly in low-resource settings. The patient’s constellation of malignancies - breast, thyroid, and renal - underscores the importance of considering hereditary cancer syndromes in individuals with multiple primary tumors, even when classic phenotypic features are absent. While genetic confirmation is ideal, clinical criteria must guide diagnosis and surveillance where access to genetic testing is limited. Multidisciplinary collaboration, patient education, and pragmatic care strategies are essential to optimize outcomes. Improving access to genetic counseling and testing remains a critical goal to ensure timely identification and tailored management of hereditary cancer syndromes like CS, especially in LMICs.
